# Preliminary safety data from a randomised trial of early versus standard timing of administration of measles-rubella vaccine in Ugandan infants

**DOI:** 10.1007/s44337-026-00649-x

**Published:** 2026-07-30

**Authors:** Gerald Businge, Natalie G. Marchevsky, Ezekiel Mupere, Sarah Kelly, Ilona Kakai, Mary Kyohere, Andrew Edielu, Fortunate Ambangira, Brenda Kakayi, Valerie Tusubira, Cleophas Komugisha, Philippa Musoke, Yama F. Mujadidi, Merijn W. Bijlsma, Kirsty Le Doare, Merryn Voysey

**Affiliations:** 1 Makerere University, Johns Hopkins University Research Collaboration (MU- JHU), Upper Mulago Hill Road, P.O. Box 23491, Kampala, Uganda; 2 Oxford Vaccine Group, Department of Paediatrics, University of Oxford, Oxford, UK; 3 Department of Paediatrics and Child Health School of Medicine College of Health Sciences, Makerere University, Kampala, Uganda; 4 City St. George’s, London School of Medicine, London, UK; 5 Department of General Paediatrics, Academic Medical Center, University of Amsterdam, Amsterdam, The Netherlands

**Keywords:** Measles, Early vaccination, Safety, High-burden settings, Reactogenicity

## Abstract

**Background:**

Measles remains a major cause of child morbidity and mortality in low-resource settings. Young infants are particularly at risk of severe disease, with many lacking protective levels of maternal antibodies by six months. Vaccination before nine months in high-burden settings may confer earlier protection; however, concerns exist about its effectiveness at this age. We present early findings on enrolment, safety, and measles infections following the administration of a registered measles-containing vaccine at six months versus nine months (MR1) with a subsequent booster (MR2) at 12 or 18 months of age.

**Methods:**

We conducted an open-label randomised controlled non-inferiority trial at four health facilities in Kampala, Uganda. Infants aged 24–28 weeks were randomly assigned to receive MR at six and 12 months (group A), nine and 18 months (group B) or six and 18 months (group C). Infants were electronically randomised in a 1:1:1 ratio using block randomisation of varying sizes, stratified by site, maternal HIV status and baseline haemoglobin level. Caretakers recorded solicited reactions on paper diary cards, with safety-related events evaluated.

**Findings:**

450 infants received MR1 and were enrolled. No differences in local or systemic post-vaccination events between the six and nine months MR1 groups were observed [Local: 21·2% versus 21·9% (*p* = 0·959), systemic: 29·2% vs. 27·7% (*p* = 0·845)]. Most local reactions were mild and within the first four days. The majority (5/6) of hospitalisations followed common childhood illnesses, with one non-vaccine-related death six months post-MR1. Sixteen laboratory-confirmed cases of measles (10) and rubella (6), and three clinical measles cases before MR1 were registered. Most measles cases (70·0%) occurred in group C, with no measles cases registered two weeks post-MR2.

**Conclusion:**

We provide further evidence on the safety of administering a measles-containing vaccine at six months of age.

*Trial registration*: The trial was registered on 31st October 2024 with Clinicaltrials.gov with the identifier NCT06667206.

**Supplementary Information:**

The online version contains supplementary material available at 10.1007/s44337-026-00649-x.

## Introduction

Measles remains a leading cause of child mortality, especially in low-resource settings (LRS), where case-fatality rates can reach 3–30% [1]. Young children under five, the malnourished (particularly vitamin A deficient), those living in overcrowded conditions, or immunocompromised individuals (e.g. advanced HIV) are most at risk [1, 2]. Complications include otitis media (5–15%), pneumonia, croup (5–10%), and in LRS, chronic diarrhoea with protein-losing enteropathy [2]. 

Global measles cases rose by over 300% between 2020 and 2023, with the WHO African Region contributing 60–70% of the global burden [3]. In East Africa, an estimated 14 million children under five live in high-incidence areas, with 8–12 million still un- or under-vaccinated [4]. In 2020, measles caused 7·5 million infections and 60,700 deaths globally—65% of which occurred in Africa [5]. During the COVID-19 pandemic, 17 large outbreaks were reported in the region, underscoring weak routine immunisation systems [6]. Measles survivors may also suffer long-term health impacts, with over 15 million disability-adjusted life years lost annually—most preventable by vaccination [7]. 

Infants under 6 months, who are not yet eligible for routine vaccination, are disproportionately affected. Though they represent just 1·6% of the global population, they accounted for 4·3% of measles cases in a 2011to 2016 epidemiological survey —most in the African Region (37·2%) [8].

Measles virus is a monotypic, single-stranded RNA virus (*Morbillivirus*) with key antigens haemagglutinin (H) and fusion (F) proteins. Lifelong immunity follows infection due to neutralising antibodies (against H) and long-lasting T-cell responses [9, 10]. Maternal antibodies usually provide passive protection for 6–9 months, but levels vary, especially in infants of vaccinated mothers who transmit fewer antibodies than those who have had natural infection [11–13]. A multicentre study found > 50% of infants had lost protective antibody levels by 6 months [12], while a meta-analysis by Ong and colleagues revealed 70% of the infants to be seronegative by the age of four months [14]. 

The WHO recommends two doses of measles-rubella (MR) vaccine. In high-incidence areas, MR1 is given at 9 months when maternal antibodies have typically waned and risk of exposure is high [6]. However, some infants may be vulnerable earlier. Administering MR1 at 6 months could bridge this gap, though the immune response may be weaker.

In Uganda, measles outbreaks remain frequent, with a concerning representation of children under 9 months of age – timing of MR1 [15–17]. The national immunisation schedule recommends MR1 at 9 months and MR2 at 18 months [18]. While earlier MR1 may yield lower antibody responses, studies suggest T-cell responses are preserved, and the second dose can boost immunity regardless of MR1 timing [19–22]. The balance lies between offering early protection and ensuring strong, lasting immunity. In comparison to the current strategy of not vaccinating this age group at all, the benefits of administration at this age are likely to be substantial.

This study evaluates protective antibody responses in children receiving early (6 months) versus standard (9 months) MR1, and early (12 months) versus standard (18 months) MR2, in a high-incidence setting. We present early findings on enrolment, safety, and measles infection events among vaccinated infants from this clinical trial.

## Methods

### Study design

BoostME is a multicentre open-label randomised controlled non-inferiority clinical trial, investigating immune responses in children given two doses of MR vaccine at different timepoints. Infants allocated to group A receive MR at 6 and 12 months, group B at 9 and 18 months, and group C at 6 and 18 months of age.

The vaccine used in the trial is the live attenuated, freeze-dried Measles and Rubella Vaccine, manufactured by Biological E. Limited, Telangana, India. The measles virus strain in the vaccine is the CAM-70 strain combined with the Wistar RA 27/3 rubella virus strain. The vaccine was provided by the Ugandan Expanded Program on Immunisation programme (UNEPI) and is the same vaccine used in the national programme. All vaccines are stored at Makerere University Johns Hopkins University Research Collaboration (MU-JHU) pharmacy at 2–8 °C in a temperature monitored refrigerator.

### Participants

Participants were infants attending the immunisation clinics at Mulago National Referral Hospital (MUL), Kisenyi Health Centre IV (KIS), Kawaala Health Centre IV (KAW) and Komamboga Health Centre III (KOM), aged 23–28 weeks at the time of screening. Infants were included in the trial only if they had received all previous infant vaccines per the Uganda Immunisation schedule, with exemption made for any potentially missed vaccinations at birth. Infants’ caretakers were required to confirm they had no plans to relocate outside the study sites’ geographical area during the study, and to provide informed consent for their infant’s participation in the trial.

The exclusion criteria included children deemed not healthy enough to be vaccinated in the opinion of the investigators, a recent family history of measles infection since the infant’s birth, previous receipt of any measles vaccination, a family history of congenital or hereditary immunodeficiency other than HIV, receipt of more than one week of immunosuppressant or immune modifying drugs, major congenital defects or serious chronic illness likely to modify immune responses or the ability to comply with the requirements of the study, a history of any neurological disorders or seizures, administration of immunoglobulins and/or any blood products since birth or planned administration during the study period and any other abnormalities or medical history that contraindicate measles vaccination.

Any child who withdrew or was discontinued from the study prior to receipt of MR1 was replaced by an additional infant. To maintain a randomised population, infants replacing the exited participants underwent the randomisation process as opposed to being directly allocated the replaced participant’s vaccination group. Recruitment ended once 450 randomised children had received their first dose of vaccine.

### Allocation and randomisation

Infants were randomised in a 1:1:1 ratio to Group A (6 and 12 months), B (9 and 18 months), and C (6 and 18 months). Block randomisation was used with randomly varying block size stratified by site (MUL, KIS, KAW, KOM), maternal HIV status (positive, negative), and infant anaemia (< 8.0 g/dl, ≥ 8.0 g/dl). Randomisation was performed electronically, with full allocation concealment, using the built-in validated Research Electronic Data Capture (REDCap) randomisation system.

### Sample size calculation

The study was powered for two primary comparisons with appropriate alpha adjustment. With 118 participants per group the study would have 90% power to determine if long term protection in those who received early vaccination (6 months) is non-inferior to those who received standard vaccination at 9 months of age, assuming 95% of participants have protective levels of antibody (> 120 mIU/mL) at 2·5 years of age in the standard group.

Similarly, the study would have 90% power to determine if the response to a booster dose at 12 months of age is non-inferior to the response at 18 months of age in those who received an early first dose at 6 months of age. These calculations used a 10% non-inferiority margin and alpha of 0·0125. Thus 354 participants were required for the full study. We aimed to recruit 450 participants to allow for at least 20% loss to follow up.

With a total sample size of 450 participants receiving at least one vaccination in the trial, the probability of observing at least 1 safety event occurring at a rate of one in 100 was approximately 99%. If no event were observed within any individual group, this would provide 95% confidence that the true incidence in the group would be no more than 2.5%.

### Data collection

Demographic, anthropometric data and clinical evaluation are performed at screening, enrolment and subsequent study follow-up visits. Blood samples for immunogenicity assessments are collected at five timepoints; prior to both the first and second measles-rubella vaccine doses, four weeks post each vaccine, and at two and half years of age (Table 1). A blood volume of 5mls is collected at each of these timepoints. Yellow fever (YF) vaccine is administered at 9 months of age, as per the routine immunisation schedule, except for infants in group B, who received it four weeks later to avoid co-administration of YF and MR vaccine.

At each vaccination visit, study participant caretakers are trained to monitor and record local and systemic reactions using a 7-day paper diary card, specifically adapted for low literacy population.

**Table 1:** Summary of study procedures

	6 months	7 months	9 months	10 months	12 months	13 months	18 months	19 months	2·5 years
Group A: 6 & 12 months	**MR1** Blood draw 1	Blood draw 2	YF		**MR2** Blood draw 3	Blood draw 4			Blood draw 5
Group B: 9 & 18 months			**MR1** Blood draw 1	YF Blood draw 2			**MR2** Blood draw 3	Blood draw 4	Blood draw 5
Group C: 6 & 18 months	**MR1** Blood draw 1	Blood draw 2	YF				**MR2** Blood draw 3	Blood draw 4	Blood draw 5

*MR* measles-rubella vaccine, *MR1*- first MR dose, *MR2* – second MR dose, *YF* Yellow Fever vaccine (Routine)

### Outcomes

#### Safety outcome measures

Local and systemic reactions were captured in the diary cards for 7 days post-vaccination. All Serious Adverse Events (SAEs) and positive measles polymerase chain reaction (PCR), measles immunoglobin M (IgM), and/or positive rubella IgM in children presenting with febrile illness and rashes were collected throughout the study.

Local and systemic reactions were graded as grade 1 (mild), grade 2 (moderate), grade 3 (severe) and grade 4 (supplementary Table 1). Adverse events were graded as grade 1–5 (supplementary Table 2). Event grading was based on the Division of AIDS (DAIDS) Table for Grading the Severity of Adult and Paediatric Adverse Events [23]. 

#### Primary outcome measures

The primary outcome measure of the study is the geometric mean and proportion of participants with protective levels of measles neutralising antibodies at 2.5 years of age. Protective antibody levels are defined as Plaque Reduction Neutralising Titres (PRNT) > 120 mIU/mL.

#### Secondary outcome measures

Secondary outcomes of the study include measles PRNT and IgG concentrations one month after the first dose in infants receiving an early (6 months) compared to standard (9 months) dose of measles-containing vaccine (MCV); infant humoral and cellular immune response to first and second doses in children with lower or higher baseline (pre-vaccination) titres; infant PRNT and IgG responses post MR1 and MR2 given for the different vaccination schedules; anti-rubella IgG; and the effect of a measles vaccination clinical trial on public perceptions of measles immunisation. Cellular responses will be assessed using an ex vivo antigen-specific T/NK-cell stimulation assay.

#### Measles and rubella cases

Infants presenting to the study site clinics with measles-like illness (any or all of cough, coryza and conjunctivitis plus a maculopapular rash with or without fever) and, with or without a history of contact with an individual with a measles-like illness in the community had a blood and oral/nasopharyngeal specimen collected. Both the respiratory and blood specimens were tested for measles nucleic material and IgM, as well as rubella IgM, at the Uganda Virus Research Institute (UVRI). This is the national measles laboratory and supports UNEPI - Ministry of Health. The laboratory is a regional WHO Measles/Rubella Reference Laboratory that also serves Comoros, Burundi, Eritrea, Ethiopia, Kenya, Tanzania, Rwanda and the Republic of South Sudan [24]. 

Infants seen at the study clinic before MR1 with a history of contact or measles-like illness, or laboratory confirmed measles, were withdrawn from the study, and replaced.

#### Statistical analysis

For this report we present baseline characteristics in all randomised infants, and safety and reactogenicity data after the first dose of MR vaccine. Analysis of SAEs up to 30 days post-first dose of vaccine and measles and rubella cases are presented descriptively in all vaccinated infants. For reactogenicity, we compared proportions of post-MR1 reactions in infants with available diary data using chi-square tests and fisher’s exact tests, where appropriate.

Clinical and/or laboratory confirmed measles and rubella cases are descriptively summarised for all randomised infants and presented according to randomised group and timing of infection. Cumulative incidence of measles cases is presented using the Kaplan-Meier method.

Data analysis was performed in R (version 4·4·3), using data extracted from the clinical database on 8 August 2025.

## Results

### Participants

**Fig. 1:**
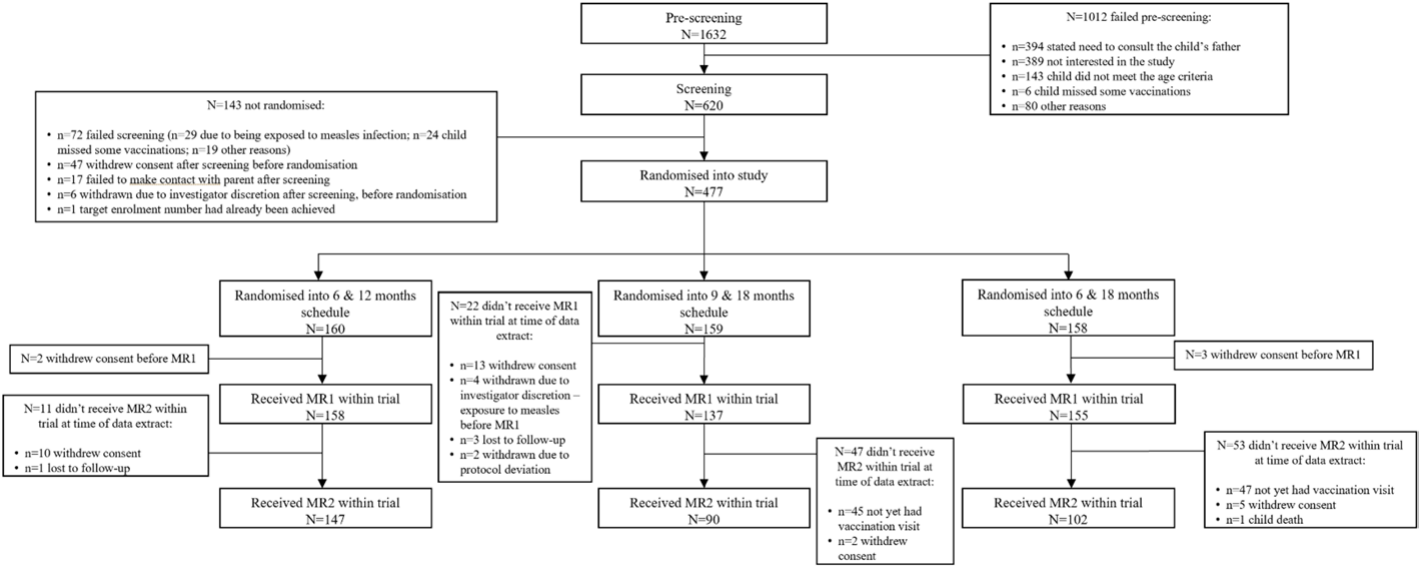
Randomisation, enrolment and vaccination flow diagram

Between 21 November 2023 and 26 September 2024, we randomised 477 participants, with 450 receiving MR1. Twenty-seven infants who were randomised but withdrawn from the trial by their caretakers or met an exclusion criterion before MR1 were replaced with a newly randomised participant (Fig. 1). More participants in group B withdrew before vaccination, in the three months between randomisation (at 6 months of age) and vaccination (at 9 months of age), than in groups A and C, where randomisation and vaccination generally occurred in the same week. Participants follow up is ongoing.

### Demographic characteristics

The median age at enrolment was 26 weeks (Interquartile range (IQR): 26–27 weeks). One hundred fifty-eight infants (35·1%) were randomised to receive MR vaccine at 6–12 months, 137 infants (30·4%) at 9–18 months and 155 infants (34·4%) at 6–18 months of age. Overall, slightly more female infants were enrolled (53·5%). Baseline characteristics were similar across randomised groups (Table 2).

**Table 2:** Baseline characteristics of randomised participants (*N* = 477)

Characteristic^1^	6 & 12 months Group A (*N* = 160)	9 & 18 months Group B (*N* = 159)	6 & 18 months Group C (*N* = 158)	Overall (*N* = 477)
Sex				
Female	90 (56.3%)	75 (47.2%)	90 (57.0%)	255 (53.5%)
Male	70 (43.8%)	84 (52.8%)	68 (43.0%)	222 (46.5%)
Age at randomisation (weeks)				
Median [Q1, Q3]	26.0 [26.0, 27.0]	26.0 [26.0, 27.0]	26.0 [26.0, 27.0]	26.0 [26.0, 27.0]
Nationality				
Ugandan	159 (99.4%)	157 (98.7%)	157 (99.4%)	473 (99.2%)
South Sudanese	0 (0%)	0 (0%)	1 (0.6%)	1 (0.2%)
Congolese	1 (0.6%)	1 (0.6%)	0 (0%)	2 (0.4%)
Pakistan	0 (0%)	1 (0.6%)	0 (0%)	1 (0.2%)
Race				
Black	160 (100%)	158 (99.4%)	158 (100%)	476 (99.8%)
Mixed Race	0 (0%)	1 (0.6%)	0 (0%)	1 (0.2%)
Haemoglobin (g/dL)	11.2 [10.4, 12.0]	11.0 [10.2, 11.8]	11.0 [10.3, 11.8]	11.0 [10.3, 11.9]
Breastfeeding	158 (98.8%)	151 (95.0%)	153 (96.8%)	462 (96.9%)
Anthropometry				
Weight at screening (kg)	7.6 [6.8, 8.2]	7.3 [6.8, 8.1]	7.4 [6.7, 8.0]	7.4 [6.8, 8.1]
Length at screening (cm)	66.0 [64.0, 67.6]	65.5 [64.0, 67.3]	65.8 [64.0, 67.5]	65.8 [64.0, 67.4]
Head Circumference at screening (cm)	43.5 [42.6, 44.5]	43.6 [42.5, 44.5]	43.4 [42.5, 44.5]	43.5 [42.5, 44.5]
Mid-Upper Arm Circumference (MUAC) at screening (cm)	14.4 [13.6, 15.2]	14.4 [13.5, 15.0]	14.4 [13.5, 15.1]	14.4 [13.5, 15.1]
Stunting^2^	17 (10.6%)	14 (8.8%)	13 (8.2%)	44 (9.2%)
Wasting^2^	1 (0.6%)	4 (2.5%)	5 (3.2%)	10 (2.1%)
Maternal HIV status ^3^				
Positive	19 (11.9%)	16 (10.1%)	17 (10.8%)	52 (10.9%)
Negative	141 (88.1%)	143 (89.9%)	141 (89.2%)	425 (89.1%)
Infant HIV status at 6 weeks of age ^4^				
Negative	18 (94·7%)	16 (100%)	17 (100%)	51 (98.1%)
Missing	1 (5·3%)	0 (0%)	0 (0%)	1 (1·9%)

^1^Median [IQR] was reported for continuous variables; n (%) for categorical variables

^2^Height/length-for-age (HAZ) and weight-for-height/length (WHZ) z-scores were calculated using the WHO Child Growth Standards. Stunting and wasting was calculated as HAZ or WHZ < -2, respectively

^3^All HIV positive mothers (52/52, 100%) were on Antiretroviral therapy at the time of infant inclusion in the trial

^4^Only infants exposed to HIV (through maternal HIV status) were tested

#### Local reactions

**Fig. 2:**
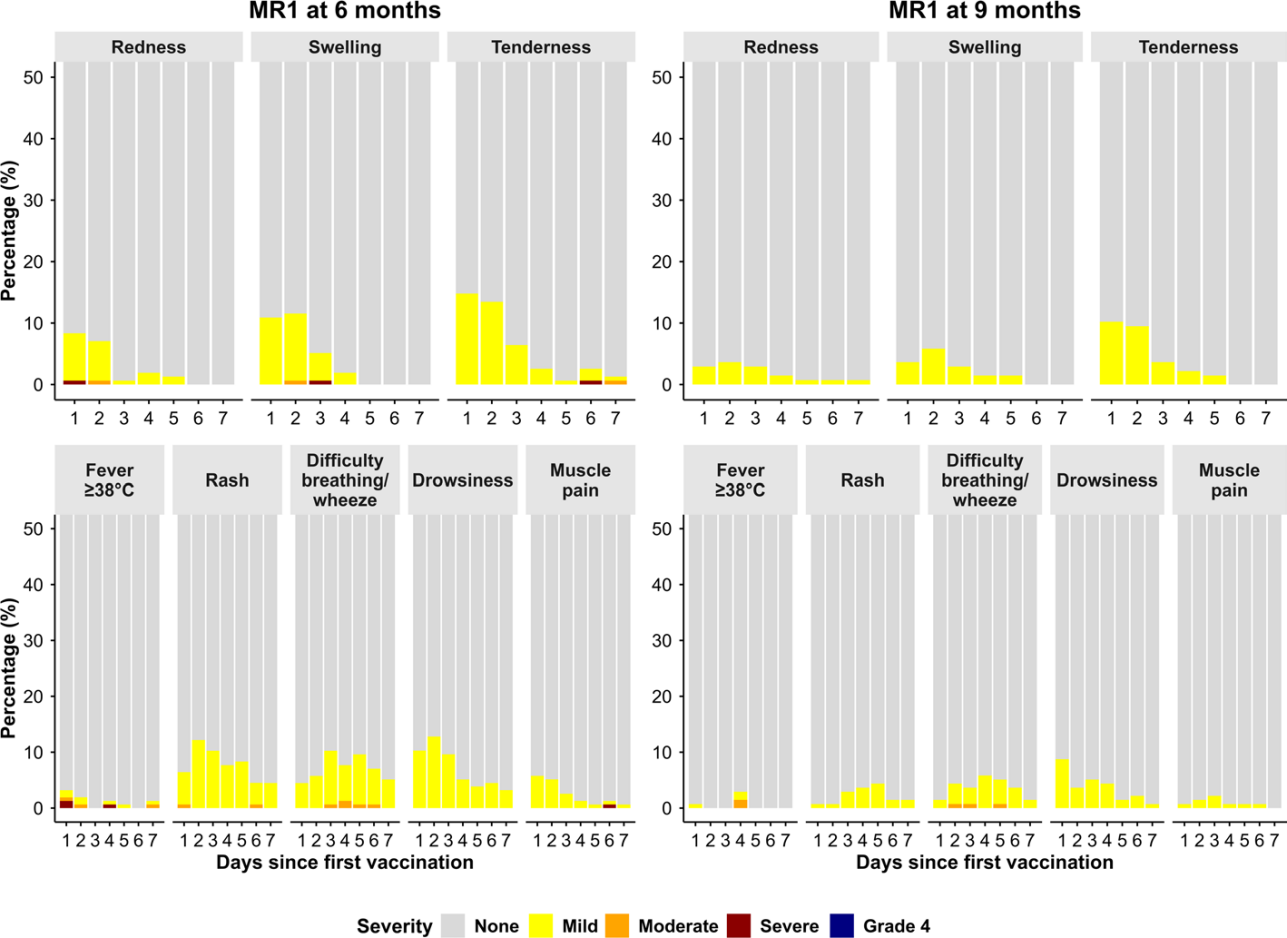
Solicited local and systemic reactions

There were no observed differences in the severity and proportion of infants experiencing local reactions after the first dose of vaccine between those vaccinated at 6 and 9 months of age. Overall, 21·2% of infants who received MR1 at 6 months registered at least one local reaction within 7 days post-vaccination compared to 21·9% of infants who received MR1 at 9 months (*p* = 0·959) (Table 3). Most local reactions were mild and occurred in the first four days post-vaccination in both 6 and 9 months arms (Fig. 2).

#### Systemic reactions

Systemic reactions were reported in similar proportions of infants receiving MR1 at 6 and 9 months (29·2% vs. 27·7%, *p* = 0·845; Table 3). Fever rates did not differ significantly (*p* = 0·781), with 1·9% of 6-month recipients and 1·5% of 9-month recipients experiencing moderate/severe fever, mostly within 48 h and on day 4, respectively (Fig. 2).

Rashes occurred in 11·8% of 6-month MR1 recipients (11·2% mild, 0·6% moderate) and 6·6% of 9-month recipients, with no significant difference (*p* = 0·125). Most rash events occurred between days 2–5 post-vaccination.

Mild to moderate wheezing or breathing difficulties were reported in 10·3% (6-month) and 10·9% (9-month) of infants (*p* = 0·957), mostly between days 2–6. Drowsiness, all mild and typically within 72 h, occurred in 13·8% of 6-month and 14·6% of 9-month recipients (*p* = 0·935).

Muscle pain was reported in 5·1% of 6-month and 2·9% of 9-month recipients (*p* = 0·456), mostly mild and within 3 days. One severe case at day 6 in a 6-month recipient resolved within 24 h.

Overall, adverse events were comparable between groups, with no observed differences across key symptoms.

**Table 3:** Solicited local and systemic reactions during days 1–7 after first vaccination

Symptom	6-month MR1 (*N* = 312)^1^	9-month MR1 (*N* = 137)	*p*-value
Any local	66 (21·2%)	30 (21·9%)	0·959
Redness	21 (6·7%)	8 (5·8%)	0·884
Swelling	28 (9·0%)	12 (8·8%)	1·000
Tenderness	39 (12·5%)	20 (14·6%)	0·650
Any systemic	91 (29·2%)	38 (27·7%)	0·845
Fever ≥ 38 °C	10 (3·2%)	5 (3·6%)	0·781^2^
Rash	37 (11·9%)	9 (6·6%)	0·125
Difficulty breathing/wheeze	32 (10·3%)	15 (10·9%)	0·957
Drowsiness	43 (13·8%)	20 (14·6%)	0·935
Muscle pain	16 (5·1%)	4 (2·9%)	0·456^2^

^1^ One participant randomised to receive MR1 at 6 months (in group A) was not included in solicited adverse event counts and proportions due to a lack of diary card data

^2^ Fisher’s exact test

Six serious adverse events (SAEs) occurred within 30 days of MR1 vaccination: four in the 9-month group and two in the 6-month group. Most (5/6) were due to infections or infestations, including diarrhoea (2 cases), malaria, pneumonia, and sepsis. One infant in the 6-month group was hospitalised for intussusception. None of the SAEs were considered related to the vaccine (Table 4).

One unrelated death from acute hydrocephalus occurred six months post-MR1 in a 12-month-old child in the 6–18 month schedule group.

**Table 4:** Summary of serious adverse events up to 30 days post-MR1

MedDRA System Organ Class Preferred Term, *n* (%)	6-month MR1 (*N* = 313)	9-month MR1 (*N* = 137)	Overall (*N* = 450)
Number of serious adverse events	2 (33·3%)	4 (66·7%)	6 (100%)
Gastrointestinal disorders	**1 (50·0%)**	**0 (0%)**	**1 (16·7%)**
Intussusception	1 (50·0%)	0 (0%)	1 (16·7%)
Infections and infestations	**1 (50·0%)**	**4 (100%)**	**5 (83·3)**
Diarrhoea infectious	0 (0%)	1 (25·0%)	1 (16·7%)
Gastroenteritis	0 (0%)	1 (25·0%)	1 (16·7%)
Malaria	0 (0%)	1 (25·0%)	1 (16·7%)
Pneumonia	1 (50·0%)	0 (0%)	1 (16·7%)
Sepsis	0 (0%)	1 (25·0%)	1 (16·7%)

*MedDRA* Medical Dictionary for Regulatory Activities

#### Measles and rubella cases

**Fig. 3:**
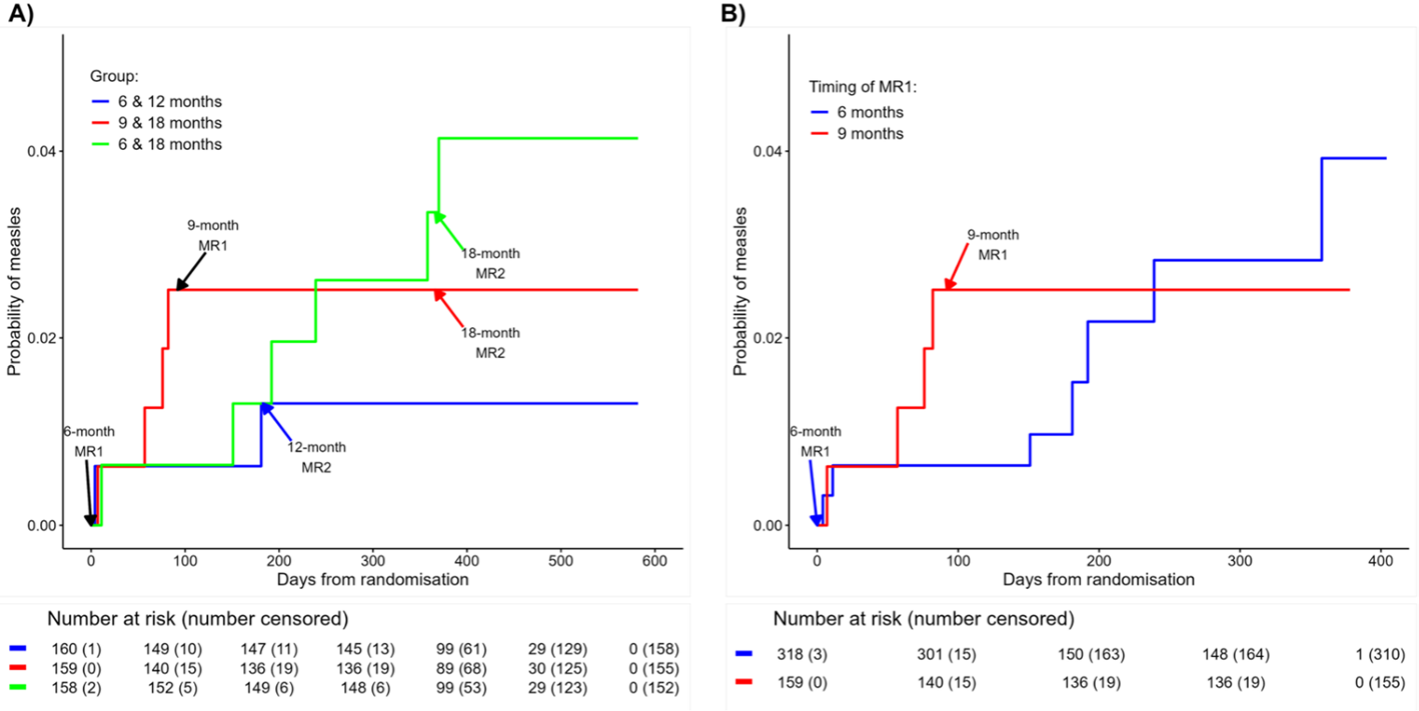
Cumulative incidence of **A** all measles cases, **B** measles cases before MR2

Sixteen laboratory-confirmed cases were detected among 72 children with morbilliform rashes: ten measles and six rubella (Table 5; Fig. 3). Three additional clinical measles cases occurred prior to MR1 without confirmatory testing and were subsequently withdrawn from further study participation. Three measles cases were in children born to HIV-positive mothers.

Of the ten confirmed measles cases, one occurred before MR1, eight between MR1 and MR2 in children vaccinated at 6 months, and one shortly after MR2. Most measles cases (7/10, 70·0%) occurred in the 6 − 18 month schedule group, likely due to the longer interval between doses. No measles cases were detected more than two weeks after MR2, though follow-up is ongoing.

Seven rubella cases were confirmed by IgM, in two infants within each vaccination group. One child in the 9-month group had two rubella episodes six months apart. Most rubella cases (6/7) occurred before MR2; one occurred 47 days post-MR2 in the 6–12 month group.

**Table 5:** Infants with diagnosed measles or rubella

Timepoint	Event		Randomised group		
			6 & 12 months (*N* = 160)	9 & 18 months (*N* = 159)	6 & 18 months (*N* = 158)
All	Measles	Clinical diagnosis only*	0 (0·0%)	3 (1·9%)	0 (0·0%)
		PCR+/IgM+	2 (1·3%)	1 (0·6%)	7 (4·4%)
	Rubella (IgM+)		2 (1·3%)	2 (1·3%)^†^	2 (1·3%)
Before MR1	Measles	Clinical diagnosis only*	0 (0·0%)	3 (1·9%)	0 (0·0%)
		PCR+/IgM+	0 (0·0%)	1 (0·6%)^€^	0 (0·0%)
	Rubella (IgM+)		0 (0·0%)	0 (0·0%)	0 (0·0%)
After MR1 before MR2	Measles (PCR+/IgM+)		2 (1·3%)**	0 (0·0%)	6 (3·8%)***^,$^
	Rubella (IgM+)		1 (0·6%)	2 (1·3%)^†^	2 (1·3%)
After MR2	Measles (PCR+/IgM+)		0 (0·0%)	0 (0·0%)	1 (0·6%)^¥^
	Rubella (IgM+)		1 (0·6%)	0 (0·0%)	0 (0·0%)

^*^ Withdrawn from the study prior to MR1 vaccination due to clinical measles diagnoses that were not laboratory-confirmed (no samples for testing)

^€^ 1 infant was exposed to HIV+ mother, taking ART during pregnancy

^$^ 2 infants were exposed to HIV+ mother, taking ART during pregnancy

^**^ 1 infant had positive measles PCR and IgM samples 4 days post-MR1 vaccination

^***^ 1 infant had positive measles PCR and IgM samples 11 days post-MR1 vaccination and a history of exposure to measles in their neighborhood. This infant died from acute hydrocephalus 198 days post-MR1 vaccination (187 days after positive measles samples), deemed not related to MR vaccination

^†^ 1 infant had 2 positive rubella IgM samples prior to MR2 vaccination: 14 and 201 days post-MR1 vaccination

^¥^ 1 infant had positive measles PCR and IgM samples 6 days post-MR2 vaccination

## Discussion

To our knowledge, this is the first randomised clinical trialto assess the safety, tolerability, and immunogenicity of early MR vaccine administration at 6 months (early prime) versus the standard 9-month schedule, as a standard two-dose regimen schedule, in a high-burden setting using a licensed vaccine. Previous work by Sayi et al. explored the immunogenicity and safety of the MR vaccine when administered at six months. The authors concluded there was no difference in the proportions of infants seroconverting between two cohorts receiving MR1 at nine months, with one cohort having received an extra MR dose at six months. Vaccination at 6 months was recommended as a supplementary dose for infants under 9 months in high transmission settings [26]. We demonstrate that MR1 at 6 months is well tolerated, with a safety profile comparable to MR1 at 9 months. However, all measles cases registered in the trial, at the time of this analysis, occurred in the 6-month group, suggesting a potential trade-off between early protection and optimal immunogenicity. The similar number of rubella cases across groups highlights the ongoing circulation of both viruses in this setting.

No differences were observed in local reactions following MR1 at 6 months (21.2%) vs. 9 months (21·9%). These findings align with prior studies from the Netherlands and Ghana, and with systematic reviews of MCV safety. In the Dutch study, local reactions ranged from 5% to 10% depending on age at vaccination (*p* = 0·08) [27]. The Ghana study reported no difference in local events between infants receiving AIK-C at 6 months and Schwarz strain at 9 months [28]. Nic Lochlainn et al. similarly reported no significant risk difference in local reactions by age [25]. Notably, local reactions may also result from injection trauma, as demonstrated by comparable local event rates after MCV or sterile water injection in young infants [29]. 

Systemic reactogenicity was also similar between groups (29·2% vs. 27·7%, *p* = 0·845), consistent with meta-analyses showing no differences in fever (0·02, 95% CI − 0·02 to 0·05) or rash (0·00, 95% CI − 0·06 to 0·06) by age [25]. The Ghana study reported higher fever in the 6-month group (21·7% vs. 11·4%, *p* = 0·01) but no overall differences in systemic events [28]. In contrast, the Dutch study observed fewer systemic events in the 6-month group, with rash the only significantly different event across ages (8% under 9 months vs. 20% at 9–11 months and 18% at 12–14 months, *p* = 0·0004). Post-vaccination reactions were mild and self-limiting, with minimal recurrence after MR2 dosing, except in rare cases of anaphylaxis [1, 30]. 

One child required surgery for intussusception, and five others were hospitalised for common infectious illnesses. None of these serious adverse events were attributed to vaccination.

Consistent with Uganda’s IDSR guidelines, children presenting with febrile maculopapular rashes were tested for measles and rubella [31]. Ten laboratory-confirmed measles cases were observed: one prior to MR1, eight post-MR1 in the 6-month group, and one post-MR2. Most (7/10, 70·0%) occurred in children following the 6–18 month schedule, suggesting that a shorter interval between MR1 and MR2 may reduce the risk of measles infection and disease. By contrast, four pre-vaccination measles cases occurred in the 9-month group during the short 3-month wait period, illustrating the vulnerability of infants in high-burden settings under current policy.

Two measles cases occurred 2–14 days post-MR1 with no known contact and were adjudicated as possibly vaccine-related. Although rash and mild illness may occur after MCV due to vaccine viral replication, true vaccine-associated measles is rare [32–35]. In a Chinese study of 15,000 genotyped cases, only 0·67% were vaccine-associated, nearly all after the first dose [36]. In the absence of sequencing, it is unclear whether post-vaccination cases in our study were wild-type or vaccine-strain. While rare severe vaccine-strain disease has been reported in immunocompromised individuals, transmission from vaccine-related cases remains largely theoretical [37, 38]. 

All measles cases in our study presented with rash and fever or coryza. As expected, these infections were mild to moderate, consistent with prior reports of milder disease in vaccinated children [1, 39, 40]. A second vaccine dose is needed to ensure protection in initial non-responders, who may represent up to 15% of children. Immunological analyses are underway to further explore this.

We also detected seven rubella events in six children, occurring in two infants in each vaccination group. One child in the 9–18 month group had two confirmed rubella episodes. Most cases (6/7) occurred after MR1 but before MR2, including one case 47 days post-MR2. Although a single rubella vaccine dose is 94–100% effective [41, 42], sporadic post-vaccination cases have been reported. In a cohort of over 77,000 children, Geier et al. reported 57 rubella cases among 33,000 vaccinated individuals, most of which were mild [43]. 

Rubella is less infectious than measles but often transmitted by asymptomatic carriers [42, 44, 45]. In our study, 4 of 6 rubella cases, including the post-MR2 case, had contact with an individual with a measles-like illness. These findings support the view that rubella cases in vaccinated children are often associated with prolonged or close exposure to infectious contacts.

Due to the small number of measles and rubella cases, our study is not powered to compare vaccine efficacy between 6- and 9-month schedules. Similarly, rubella cases were too few to draw firm conclusions on schedule effectiveness. Nevertheless, high community coverage (≥ 95%) remains critical to interrupt transmission of both viruses.

### Strengths and limitations

The diagnosis of measles/rubella requires keen clinician observation and a high index of suspicion. While children presenting to the study clinics with a measles-like illness were investigated, we acknowledge that some children might have sought medical attention at other health care facilities outside the study sites and therefore were not captured. Second, we were unable to determine at the time of presentation if the registered measles cases were due to primary or secondary vaccine failure. We were equally unable to obtain measles/rubella genetic characterisation to rule out the possibility of a vaccine strain in the measles/rubella cases recorded after vaccination.

Despite these limitations, the study design has adequately enabled the recording and follow-up of all adverse events, including measles/rubella events, across the three vaccination groups for an extended observation timeline. The pre- and post-vaccination sample collection schedule provides a valuable opportunity in assessing the short- and long-term immunological response following an early and standard measles and rubella vaccination schedule. Additionally, we will be able to determine the amount and potential influence of pre-vaccination measles antibodies in Ugandan infants.

### Implications of results and conclusion

This study provides further evidence supporting the safety of administering a measles-containing vaccine at 6 months of age. The occurrence of measles and rubella cases in children who had received at least one vaccine dose underscores the continued widespread transmission of both viruses among Ugandan infants and highlights the urgent need to achieve and maintain high two-dose vaccine coverage.

Although all measles cases occurred in children who received MR1 at 6 months, the number of cases is too small to draw definitive conclusions about differences in vaccine efficacy between schedules. Immunogenicity analyses will help determine whether these cases occurred in vaccine non-responders. An extension of the trial is underway to increase sample size and evaluate the clinical impact of vaccine timing more robustly.

## Supplementary Information

Below is the link to the electronic supplementary material.

Supplementary Material 1.

Supplementary Material 2.

Supplementary Material 3.

Supplementary Material 4.

Supplementary Material 5.

Supplementary Material 6.

Supplementary Material 7.

Supplementary Material 8.

## Data Availability

The demographic and clinical evaluation data analysed and presented in this report are available as Supplementary Files.
