# Supplementary material for: Preliminary safety data from a randomised trial of early versus standard timing of administration of measles-rubella vaccine in Ugandan infants
Source: Discov Med (Singap). 2026 Jul 30;3(1):92. doi: 10.1007/s44337-026-00649-x (PMC13424465; doi:10.1007/s44337-026-00649-x)
Supplement: Supplementary file 7 — Supplementary Material 7. [file 44337_2026_649_MOESM7_ESM.pptx]

## Slide 1
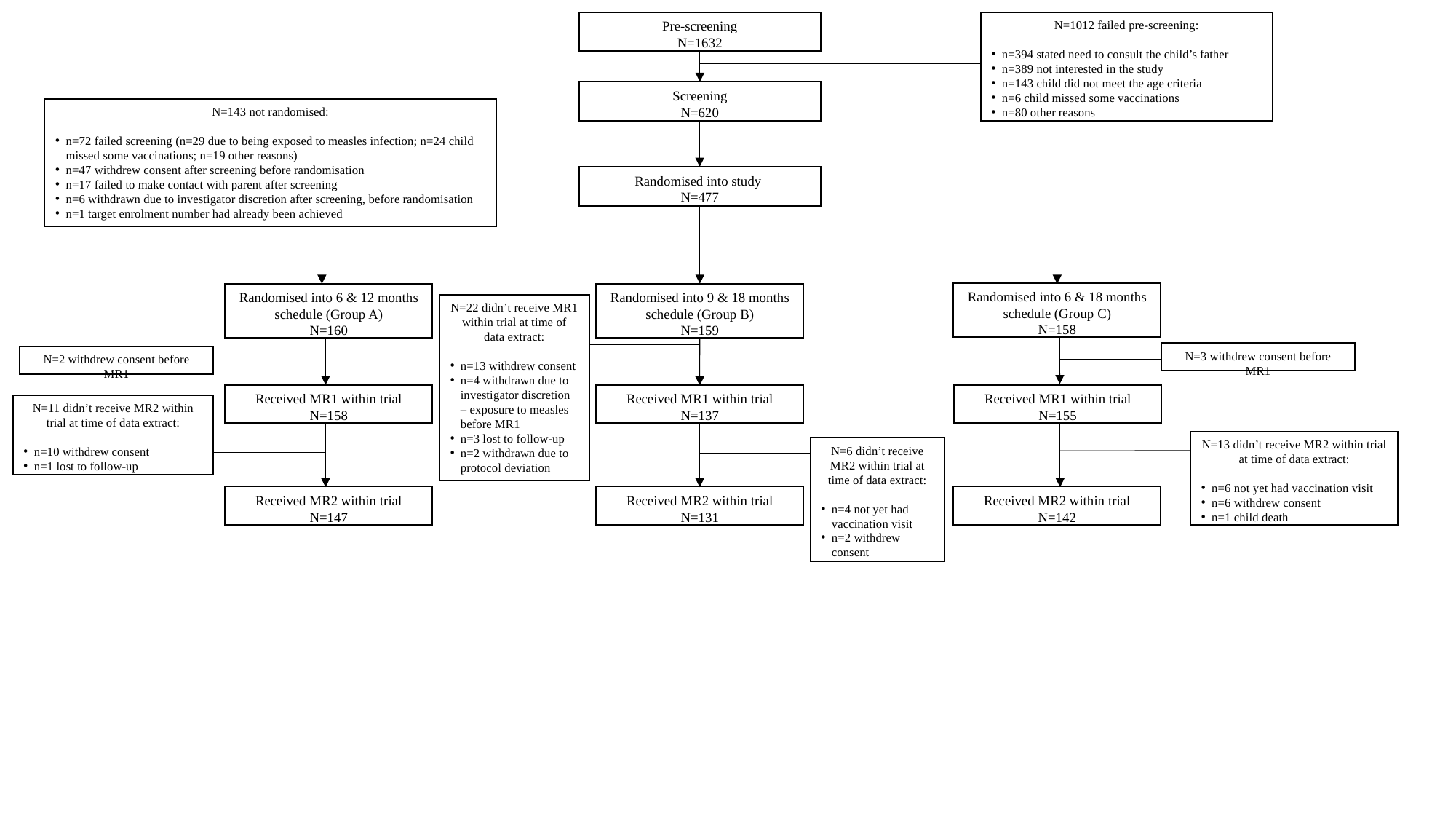

Pre-screening
N=1632
N=1012 failed pre-screening:
n=394 stated need to consult the child’s father
n=389 not interested in the study
n=143 child did not meet the age criteria
n=6 child missed some vaccinations
n=80 other reasons
Screening
N=620
N=143 not randomised:
n=72 failed screening (n=29 due to being exposed to measles infection; n=24 child missed some vaccinations; n=19 other reasons)
n=47 withdrew consent after screening before randomisation
n=17 failed to make contact with parent after screening
n=6 withdrawn due to investigator discretion after screening, before randomisation
n=1 target enrolment number had already been achieved
Randomised into study
N=477
Randomised into 6 & 18 months schedule (Group C)
N=158
Randomised into 6 & 12 months schedule (Group A)
N=160
Randomised into 9 & 18 months schedule (Group B)
N=159
N=22 didn’t receive MR1 within trial at time of data extract:
n=13 withdrew consent
n=4 withdrawn due to investigator discretion – exposure to measles before MR1
n=3 lost to follow-up
n=2 withdrawn due to protocol deviation
N=3 withdrew consent before MR1
N=2 withdrew consent before MR1
Received MR1 within trial
N=158
Received MR1 within trial
N=137
Received MR1 within trial
N=155
N=11 didn’t receive MR2 within trial at time of data extract:
n=10 withdrew consent
n=1 lost to follow-up
N=13 didn’t receive MR2 within trial at time of data extract:
n=6 not yet had vaccination visit
n=6 withdrew consent
n=1 child death
N=6 didn’t receive MR2 within trial at time of data extract:
n=4 not yet had vaccination visit
n=2 withdrew consent
Received MR2 within trial
N=147
Received MR2 within trial
N=131
Received MR2 within trial
N=142
