# Supplementary material for: Preliminary safety data from a randomised trial of early versus standard timing of administration of measles-rubella vaccine in Ugandan infants
Source: Discov Med (Singap). 2026 Jul 30;3(1):92. doi: 10.1007/s44337-026-00649-x (PMC13424465; doi:10.1007/s44337-026-00649-x)
Supplement: Supplementary file 8 — Supplementary Material 8. [file 44337_2026_649_MOESM8_ESM.docx]

Supplementary Table 1 Local and Systemic event grading

| **Signs and Symptoms** | **Mild (Grade 1)** | **Moderate (Grade 2)** | **Severe (Grade 3)** | **Grade 4** |
| --- | --- | --- | --- | --- |
| Tenderness | Does not interfere with activity | Repeated use of non-narcotic pain reliever >24 hours or interferes with activity | Any use of narcotic pain reliever or prevents daily activity | Emergency room visit or hospitalisation |
| Redness | 2·5 cm to 5·0 cm  (5 to 10 measuring device units) | >5·0 cm to 10·0 cm  (11 to 20 measuring device units) | >10cm  (21 measuring device units) | Necrosis or exfoliative dermatitis |
| Swelling | 2·5 cm to 5·0 cm  (5 to 10 measuring device units) | >5·0 cm to 10·0 cm  (11 to 20 measuring device units) | >10cm  (21 measuring device units) | Necrosis |
| Temperature | 38·0°C to 38·4°C (100·4°F to 101·1°F) | 38·0°C to 38·4°C (100·4°F to 101·1°F) | >38·9°C to 40·0°C (102·1°F to 104·0°F) | >40·0°C (>104·0°F) |
| Rash | Small number of hives, limited localised rash | Widespread rash | Evolving Stephen’s Johnson syndrome | Emergency room visit, shock or hypotension |
| Difficulty breathing/wheeze | Some increase in work of breathing, minimal cough | Moderate work of breathing, constant cough/wheeze | Severe work of breathing, intercostal and subcostal recession. Breathless. Oxygen saturations <92% in air | Emergency room visit or hospitalisation |
| Drowsiness | No interference with activity | Some interference with activity | Significant; prevents daily activity | Emergency room visit or hospitalization |
| Muscle pain | No interference with activity | Some interference with activity | Significant; prevents daily activity | Emergency room visit or hospitalization |

Supplementary Table 2 Adverse Events severity grading

| **Grade** | **Severity** | **Definition** |
| --- | --- | --- |
| Grade 1 | Mild | asymptomatic or mild symptoms; no or minimal interference with usual social & functional activities, intervention not indicated |
| Grade 2 | Moderate | moderate symptoms causing greater than minimal interference with usual social & functional activities, intervention indicated |
| Grade 3 | Severe | severe symptoms causing inability to perform usual social & functional activities with intervention or hospitalisation indicated |
| Grade 4 | Potentially Life-Threatening | Symptoms causing inability to perform basic self-care functions with intervention indicated to prevent permanent impairment, persistent disability or death |
| Grade 5 | Death | Death |

Supplementary Table 3 Baseline characteristics of vaccinated participants

| **Characteristic^1^** | **6 & 12 months**  **Group A N = 158^1^** | **9 & 18 months**  **Group B N = 137^1^** | **6 & 18 months**  **Group C N = 155^1^** | **Overall**  **N = 450^1^** |
| --- | --- | --- | --- | --- |
| **Sex** |  |  |  |  |
| Female | 90 (57.0%) | 64 (46.7%) | 88 (56.8%) | 242 (53.8%) |
| Male | 68 (43.0%) | 73 (53.3%) | 67 (43.2%) | 208 (46.2%) |
| **Age at randomisation (weeks)** | 26.0 [26.0, 27.0] | 26.0 [26.0, 27.0] | 26.0 [26.0, 27.0] | 26.0 [26.0, 27.0] |
| **Nationality** |  |  |  |  |
| Ugandan | 157 (99.4%) | 135 (98.5%) | 154 (99.4%) | 446 (99.1%) |
| South Sudanese | 0 (0%) | 0 (0%) | 1. (0.6%) | 1 (0.2%) |
| Congolese | 1 (0.6%) | 1 (0.7%) | 0 (0%) | 2 (0.4%) |
| Pakistan | 0 (0%) | 1 (0.7%) | 0 (0%) | 1 (0.2%) |
| **Race** |  |  |  |  |
| Black | 158 (100%) | 136 (99.3%) | 155 (100%) | 449 (99.8%) |
| Mixed Race | 0 (0%) | 1 (0.7%) | 0 (0%) | 1 (0.2%) |
| **Haemoglobin (g/dL)** | 11.2 [10.3, 12.0] | 10.9 [10.2, 11.9] | 11.0 [10.2, 11.8] | 11.0 [10.2, 11.9] |
| **Breastfeeding** | 157 (99.4%) | 130 (94.9%) | 150 (96.8%) | 437 (97.1%) |
| **Anthropometry** |  |  |  |  |
| Weight at screening (kg) | 7.6 [6.8, 8.2] | 7.4 [6.8, 8.1] | 7.4 [6.7, 8.0] | 7.4 [6.8, 8.1] |
| Length at screening (cm) | 66.0 [64.0, 67.6] | 65.3 [64.0, 67.0] | 65.8 [64.0, 67.5] | 65.8 [64.0, 67.4] |
| Head Circumference at screening (cm) | 43.5 [42.6, 44.5] | 43.6 [42.5, 44.5] | 43.4 [42.5, 44.5] | 43.5 [42.5, 44.5] |
| MUAC at screening (cm) | 14.4 [13.5, 15.1] | 14.4 [13.5, 15.0] | 14.3 [13.5, 15.1] | 14.4 [13.5, 15.0] |
| **Maternal HIV status** |  |  |  |  |
| Positive | 19 (12.0%) | 12 (8.8%) | 17 (11.0%) | 48 (10.7%) |
| Negative | 139 (88.0%) | 125 (91.2%) | 138 (89.0%) | 402 (89.3%) |
| **Infant HIV status at 6 weeks of age^2^** |  |  |  |  |
| Negative | 18 (94·7%) | 12 (100%) | 17 (100%) | 47 (97.9%) |
| Missing | 1 (5.3%) | 0 (0%) | 0 (0%) | 1 (2·1%) |

*^1^* Median [IQR] was reported for continuous; n (%) for categorical variables

*^2^* Only infants exposed to HIV (through maternal HIV status) were tested
